# Theoretical prediction of two-dimensional BC_2_X (X = N, P, As) monolayers: ab initio investigations

**DOI:** 10.1038/s41598-022-26805-8

**Published:** 2022-12-23

**Authors:** A. Bafekry, M. Naseri, M. Faraji, M. M. Fadlallah, D. M. Hoat, H. R. Jappor, M. Ghergherehchi, D. Gogova, H. Afarideh

**Affiliations:** 1 Department of Physics and Energy Engineering, Amirkabir University of Technology, Tehran, Iran; 2 Department of Physics, University of Guilan, Rasht, 41335-1914 Iran; 3 Department of Chemistry, Department of Physics and Astronomy, CMS-Center for Molecular Simulation, Institute for Quantum Science and Technology, Quantum Alberta, University of Calgary, 2500 University Drive NW, Calgary, AB T2N 1N4 Canada; 4 Micro and Nanotechnology Graduate Program, TOBB University of Economics and Technology, Sogutozu Caddesi No 43 Sogutozu, 06560 Ankara, Turkey; 5 Department of Physics, Faculty of Science, Benha University, Benha, 13518 Egypt; 6 Institute of Theoretical and Applied Research, Duy Tan University, Ha Noi, 100000 Viet Nam; 7 Faculty of Natural Sciences, Duy Tan University, Da Nang, 550000 Viet Nam; 8 Department of Physics, College of Education for Pure Sciences, University of Babylon, Hilla, Iraq; 9 Department of Electrical and Computer Engineering, Sungkyunkwan University, Suwon, 16419 Korea; 10 Department of Physics, Chemistry and Biology, Linkoping University, 58183 Linköping, Sweden

**Keywords:** Nanoscale materials, Materials for devices, Nanoscale materials

## Abstract

In this work, novel two-dimensional BC$_2$X (X = N, P, As) monolayers with X atoms out of the B–C plane, are predicted by means of the density functional theory. The structural, electronic, optical, photocatalytic and thermoelectric properties of the BC$_2$X monolayers have been investigated. Stability evaluation of the BC$_2$X single-layers is carried out by phonon dispersion, ab-initio molecular dynamics (AIMD) simulation, elastic stability, and cohesive energies study. The mechanical properties reveal all monolayers considered are stable and have brittle nature. The band structure calculations using the HSE06 functional reveal that the BC$_2$N, BC$_2$P and BC$_2$As are semiconducting monolayers with indirect bandgaps of 2.68 eV, 1.77 eV and 1.21 eV, respectively. The absorption spectra demonstrate large absorption coefficients of the BC$_2$X monolayers in the ultraviolet range of electromagnetic spectrum. Furthermore, we disclose the BC$_2$N and BC$_2$P monolayers are potentially good candidates for photocatalytic water splitting. The electrical conductivity of BC$_2$X is very small and slightly increases by raising the temperature. Electron doping may yield greater electric productivity of the studied monolayers than hole doping, as indicated by the larger power factor in the n-doped region compared to the p-type region. These results suggest that BC$_2$X (X = N, P, As) monolayers represent a new promising class of 2DMs for electronic, optical and energy conversion systems.

## Introduction

The discovery of graphene has motivated enormous theoretical and experimental endeavors in two-dimensional (2D), van der Waals (vdW) materials study^1^, for attaining new properties and designing of innovative devices. A large number of 2D materials have been tested including elemental semiconductors (phosphorene, stanene, silicene, borophene, antimonene, germanene)^2^, carbon nitrides^3^, indium and gallium chalcogenide (GaS, InSe, GaSe)^4^, transition metal chalcogenide (FeSe, FeTe, CdS, CdSe, MoSe$_{2}$)^5–7^, pnictogens-based monolayer (Sb$_{2}$X$_{3}$ (X = S, Se, Te))^8^, transition metal oxides (ZnO$_{2}$, CdO$_{2}$ HgO$_{2}$)^9^, and Janus materials.

Potential applications in almost all fields of modern technology, such as energy^10^ storage sensors, field-effect transistors, spintronics, photocatalytic hydrogen production, catalysis, and supercapacitors^11–13^ have been demonstrated. In latest years, 2D materials based on boron element have drawn a lot of interest due to its exceptional layer structure and excellent electronic, optical and mechanical properties. Prompted by such fascinating properties, the boron-based 2D materials potential can be used in thermoelectric applications^14^, battery applications^15^, clean energy storage^16^, water cleaning^17^, sensing^18^, photovoltaic^19^, and many more. In particular, as the front runner in boron-carbon materials family, a compound of BC shows a large surface-to-volume ratio, tunable bandgap, high thermal stability, numerous active sites, and multilateral bonding of both carbon and boron atoms, making it an excellent candidate for optoelectronics applications. Besides, carbon and boron can indeed be mixed to produce a variety of 2D materials, however, some of them have stable and strong covalent bonds^20,21^.

Among the number of detailed investigations, both experimental and theoretical, studies focused mainly on BCN monolayer, composed of boron, carbon, and nitrogen atoms^22–24^. The proposed new type of semiconductor monolayer (BCN) exhibited a range of band structures from semiconducting to insulating, based on their geometry and composition^25^ as well as high carrier mobility and absorption in the visible range^26^. Thus, it proffers a variety of applications in catalysis, hydrogen evolution reaction, electrochemical sensors, transistors, electrochemical energy storage devices, and nanoelectronics^27–31^. On the other hand, 2D BCN monolayer has been experimentally fabricated^32^. Furthermore, other authors expected that free-standing monolayers can be produced by peeling because of the extreme intermolecular bonding between the atoms within the monolayer^33^. Zhang Li et al. have found by MD simulations that the hexagonal graphene-like BCN monolayer exhibits a lower thermal conductivity than graphene^34^. However, as has been exposed in the work of Zhang et al., BCN monolayer has a fracture strength value (81.4–93.5 GPa) lower than that of graphene and the temperature has a sturdy impact on its mechanical properties^35^. In this context, Thomas and Lee predicted that the BCN monolayer has perfect mechanical properties with higher directional anisotropy, higher flexibility and Young’s modulus lesser than that of graphene^36^. Furthermore, the 2D BC$_{2}$N monolayer, with all atoms in one plane, has been synthesized. The experimental results show that BC$_{2}$N monolayer is a semiconductor with a direct bandgap of $\sim$ 2 eV^37^. Its electronic and thermal properties have been studied. The final conclusion was that BC$_{2}$N monolayer is a good candidate for thermoelectric devices^38,39^. Also, the BC$_{2}$N monolayer can be a suitable anode in Li- and Na-ion batteries^40^. Considering the fact that nitrogen can enhance the electronic, thermal, optical, and mechanical properties, it is interesting to study the impact of phosphorus (P) and arsenic (As) neighboring N in the nitrogen (pnictogen) group on these properties.

In line with the ongoing efforts made in the field of hexagonal 2D structures, in the present study, we propose and systematically investigate for the first time the structural, electronic structure, optical and thermoelectric properties of three kinds of a graphene-like BC$_2$X (X = N, P, As) monolayers, with X atoms out of the plane of the C and B atoms. Remarkably, the intermixture of BC and N, P, and As atoms can dramatically change the electronic structure, enhance the dynamic stability of various 2D materials, and may lead to BC$_2$X with bandgaps in the visible range. Our simulation results provide some guidelines for future experimental studies on graphene-like BC$_2$X monolayers.

## Method

The density-functional theory calculations were performed by using the plane-wave basis projector augmented wave and the generalized gradient approximation of Perdew–Burke–Ernzerhof (PBE)^41,42^ as implemented in the VASP package^43,44^. The band structure calculations were obtained by the screened-nonlocal-exchange functional (HSE06)^45^ for a more accurate description. The kinetic energy cut-off was 520 eV, the variation in the energies was below 10$^{-5}$ eV and the total Hellmann–Feynman forces were less than 0.05 eV/Å for the optimized structures. The distance between two neighboring sheet, in z direction, was 20 Å, which was sufficient to avoid any image interaction. The **k**-point mesh was $20\times 20\times 1$ Monkhorst–Pack scheme^46^ for the primitive unit cell and the charge transfer analysis was performed using the Bader technique^47^. The vibrational properties were calculated as implemented in the PHONOPY code^48^. The optical properties such as dielectric constants, absorption spectra, and reflectivity were studied on the basis of the HSE06 functional. The training set is prepared by conducting ab-initio molecular dynamics (AIMD) simulations over $4\times 4\times 1$ supercells with $9\times 9\times 1$ k-point grids. AIMD simulations are carried out at 300 K.

## Structural and energetic properties

**Figure 1:**
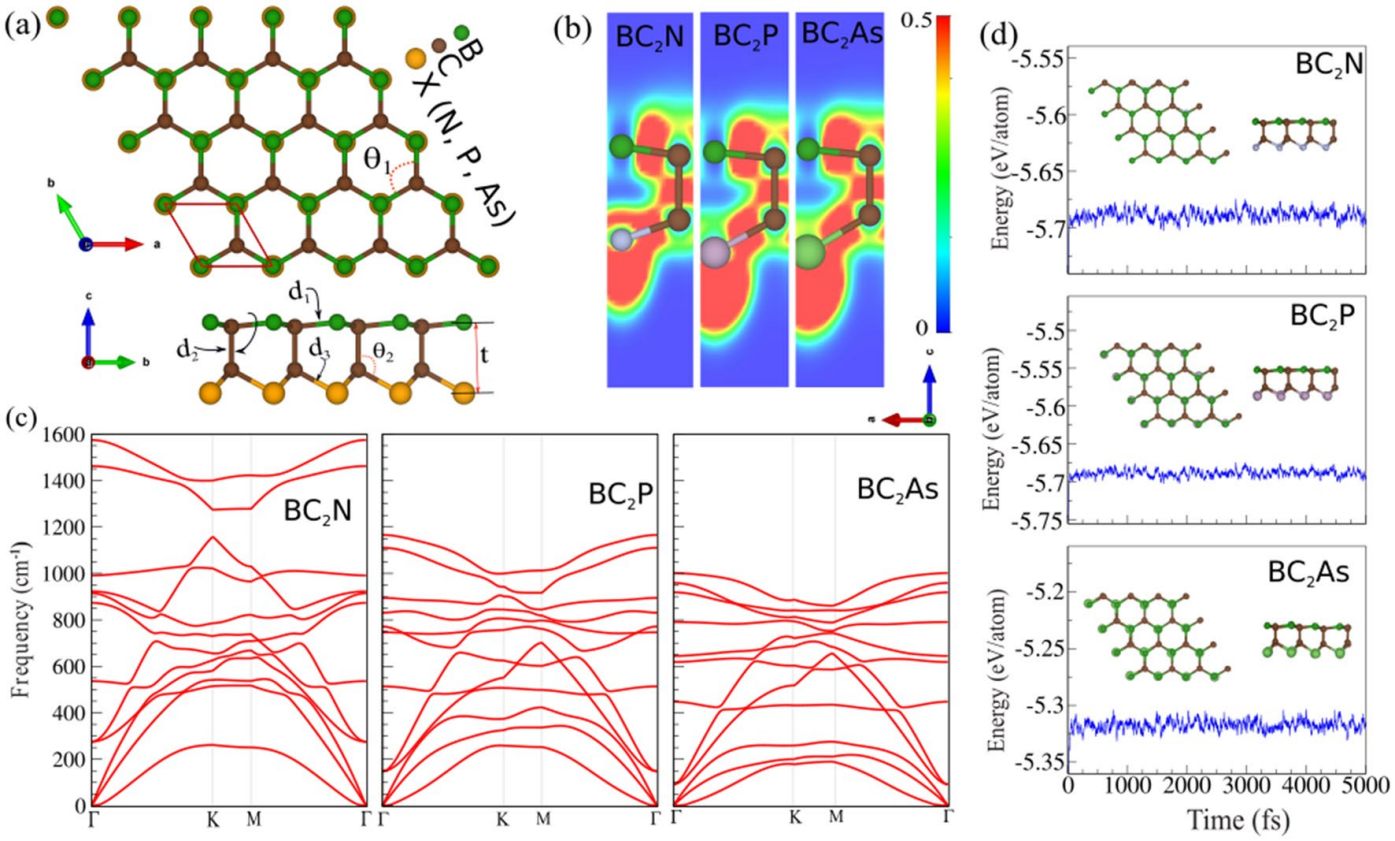
(**a**) Top and side views of monolayer structure, (**b**) contour plot of the electron localization function (ELF), (**c**) phonon dispersions and (**d**) Ab initio molecular dynamics (AIMD) 300 K temperature for the BC$_2$X (X = N, P, As) monolayers. The primitive unit cell (red parallelogram) and the structural parameters are illustrated. Red (blue) color refers to the high (low) electron density in the ELF. The structure after 5 ps of simulation demonstrated as insets in the AIMD.

**Table 1:** Structural and electronic parameters of BC$_2$X (X = N, P, As) monolayers as shown in Fig. 1a,b including lattice constants $\textit{a}$; bond lengths between B–C, C–C and C–X atoms ($\textit{d}_{1,2,3}$); thickness defined by the difference between the largest and smallest z coordinates of B and X atoms (*t*); bond angles between B–C–B and C–X–C atoms $\theta _{1,2}$; cohesive energy per atom, $(E_{coh})$; charge transfer $(\Delta {Q})$ between atoms B and *X* atoms; work function $(\Phi )$ bandgap $(E_{g})$ of PBE (HSE06); bulk modulus (B); shear modulus (S); Young’s modulus (Y); Poisson’s ratio ($\nu )$; and bulk/shear ratio (B/S), respectively.

	*a* (Å)	*d*$_{1,2,3}$ (Å)	*t* (Å)	$\theta _{1,2}$ (°)	$E_{coh}$ (Å)	$\Delta {Q}$ (eV/atom)	$\Phi$ (e)	$E_{g}$ (eV)	B (GPa)	S (GPa)	Y (GPa)	$\nu$	BS
BC$_2$N	2.56	1.49, 1.65, 1.56	2.41	117, 109	− 6.40	0.70	4.32	1.51 (2.68)	14.63	9.20	22.81	0.24	1.59
BC$_2$P	2.82	1.63, 1.65, 1.86	2.73	118, 119	− 6.16	0.80	4.00	0.78 (1.77)	4.16	3.11	7.42	0.20	1.35
BC$_2$As	2.91	1.68, 1.62, 1.95	2.79	118, 120	− 5.77	1.10	3.50	0.39 (1.21)	3.54	2.77	6.60	0.19	1.28

The top and side figures of the structure of the BC$_2$X (X = N, P, As) monolayers are shown in Fig. 1a. The hexagonal primitive unit cell with *P*3*m*1 space group is formed by four atoms and indicated by a parallelogram. The structural parameters are also defined in the Fig. 1a. We found that the lattice parameters of the BC$_2$N, BC$_2$P and BC$_2$As sheets are 2.56 Å, 2.82 Å and 2.91 Å, respectively. The calculated bond lengths of B–C ($d_{1}$), C–C ($d_{2}$) and C–X ($d_{3}$) are respectively: 1.49, 1.65 and 1.56 Å (BC$_2$N), 1.63 Å, 1.65 Å and 1.86 Å (BC$_2$P) and in the case of BC$_2$As they are 1.68 Å, 1.62 Å, 1.95 Å. The bond lengths and angles, and thickness are tabulated (see Table 1). The two angles of B–C–B and C–X–C are determined to be 117$^{\circ }$, 109$^{\circ }$ (BC$_2$N), 118$^{\circ }$, 119$^{\circ }$ (BC$_2$P) and 118$^{\circ }$, 120$^{\circ }$ (BC$_2$As). Notice that the studied monolayers are high anisotropic lattice, while the thickness of the BC$_2$N, BC$_2$P and BC$_2$As are 2.41 Å, 2.73 Å and 2.79 Å, respectively. We found that the bond lengths, bond angles and thickness increase as the atomic radius of X atom increases (see Table 1).

The electron localization function (ELF) considered monolayers is illustrated in Fig. 1b. The red (blue) color indicates high (low) electron density in the ELF. Obtained results demonstrate that there is a charge transfer from B/C to X atoms in BC$_2$X with a corresponding amount of 0.702*e*, 0.80*e*, and 1.10*e* for N, P, and As, respectively. The B/C atoms are positively charged ions that surround the N, P and As negatively charged ions. Before investigation of the electro-optical properties and possible applications of the designed 2D monolayers, the stability of the materials was analyzed. At first the energetic stability of the designed 2D monolayer materials were assessed. To reach this aim, the cohesive energy per atom of the materials was calculated by using the following formula:

1 $$\begin{aligned} E_{coh} = (E_{tot}-E_{{\textrm{B}}}-2E_{{\textrm{C}}}-E_{{\textrm{X}}})/{4}, \end{aligned}$$

where $E_{tot}$ represents the total energy of the BC$_2$X monolayers. $E_{{\textrm{B}}}$, $E_{{\textrm{C}}}$ and $E_{{\textrm{X}}}$ represent the total energies of isolated single B, C and X (N, P and As) atoms, respectively and 4 represents the total number of atoms in the primitive cell.

Our results show that the cohesive energies are − 6.40 eV/atom (BC$_2$N), − 6.16 eV/atom (BC$_2$P) and − 5.77 eV/atom (BC$_2$As). The more negative value of the cohesive energies suggests the more energetically stable monolayer is BC$_2$N. It can be seen that the structure with a smaller atomic mass or atomic radius, and high electronegativity has the greater magnitude of cohesive energy, specifically; BC$_2$N has a higher electronegativity with that of N (3.04) as compared to the electronegativity of P (2.19) and As (2.18). Although the relative stability of different atomic compositions cannot be determined directly by their cohesive energies, but the high value of obtained cohesive energies of these designed materials can be considered as a good evidences for their strong bonding properties. However, based on the calculated cohesive energies, we further considered following hypothetical reactions as possible synthesis strategy as well as a confirmation for the stability of the materials:

- Borophene + 2T-Carbon + 1/2 N$_2$ $\rightarrow$ BC$_2$N monolayer H = − 880 meV (per BC$_2$N unit),
- Borophene + 2T-Carbon + Phosphorene $\rightarrow$ BC$_2$P monolayer H = − 380 meV (per BC$_2$P unit),
- Borophene + 2T-Carbon + Arsenene $\rightarrow$ BC$_2$As monolayer H = − 140 meV (per BC$_2$N unit),

In which the energies of brophene, T-Carbon, phosphorene and arsenene are calculated by using same method of theory. It can be seen that B$_2$CN can be obtained by using Borophene monolayer, T-Carbon and nitrogen gas as reactants. Also BC$_2$P/As monolayer may be obtained by using Borophene monolayer, T-carbon and phosphorene/arsenene monolayer as reactant materials. It is worth pointing out that all of these reactant materials have been experimentally obtained.

## Structural stability and mechanical properties

The phonon dispersion of BC$_2$X (X = N, P, As) monolayers are also calculated to evaluate their dynamical stability. The phonon bands of the considered monolayers is illustrated in Fig. 1c. Obviously, the phonon branches do not contain imaginary frequencies which confirms the dynamical stability of the studied monolayers. It is shown that each monolayer material exhibits a quadratic dispersion for the ZA phonon branch around the $\Gamma$ point as a direct consequence of the 2D nature of the structure. The highest optical phonon branches are found to be at frequencies approximately 1600, 1150, and 1000$^{-1}$ for BC$_2$N, BC$_2$P, and BC$_2$As, respectively. This indicates that as the atomic mass increases from N to As, the frequency of vibrations decreases and the material gets quite softer. Moreover, the phonon gap between the highest two optical branches and the other phonons decreases as going from N to As. We then examine the thermal stability of BC$_2$X monolayers by evaluating ab initio molecular dynamics (AIMD) trajectories at 300 K. The AIMD simulations of BC$_2$X monolayers at 300 K temperature are shown in Fig. 1d. The different views of the optimized structure after 5 ps of simulation demonstrated in the insets. Analysis of the AIMD trajectories also reveals that all the structures could stay intact at 300 K with very stable temperature and energy profiles, proving the thermal stability of the BC$_2$X monolayers. This finding will definitely stimulate efforts in experimental synthesis of 2D BC$_2$X monolayers.

**Figure 2:**
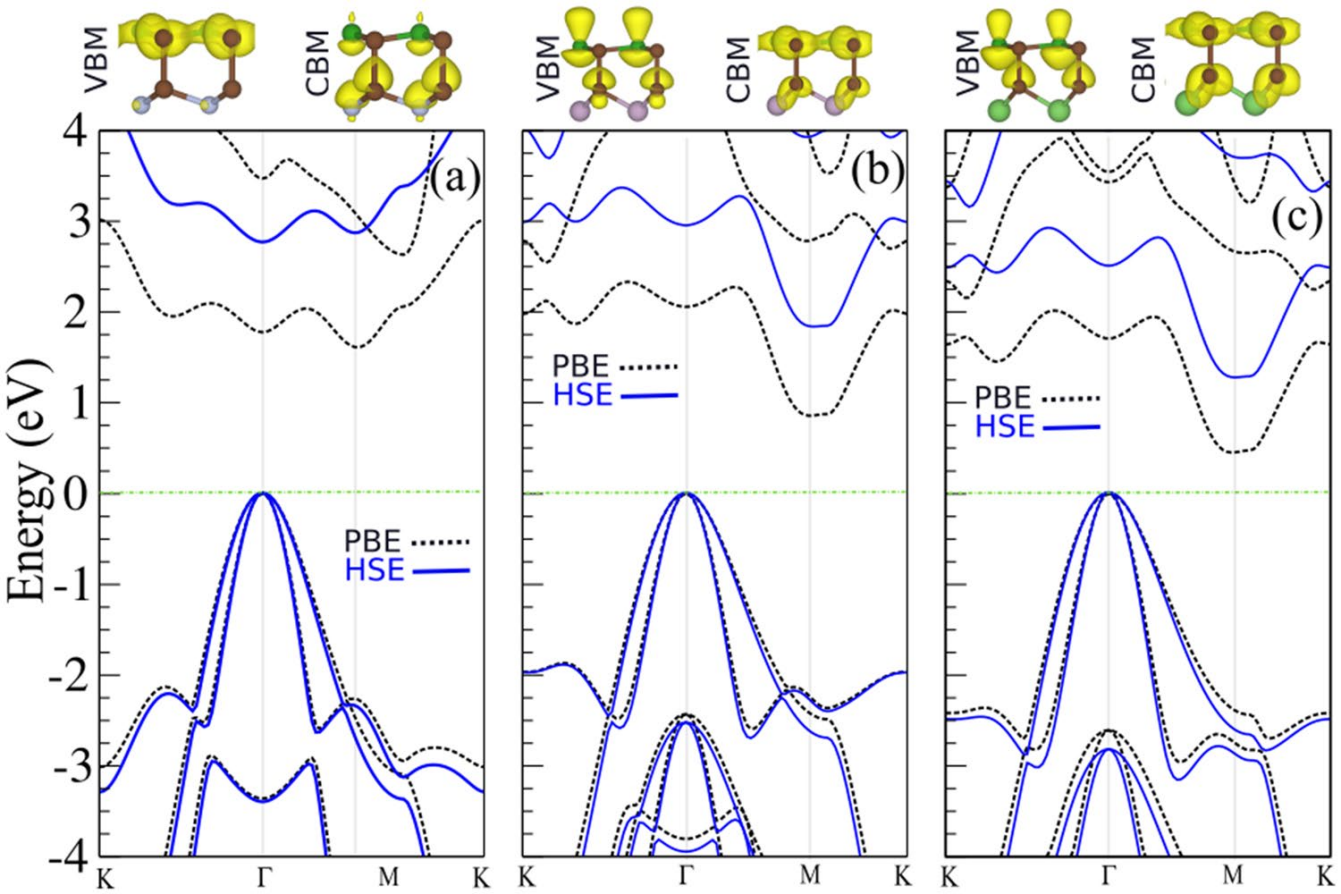
Electronic band structure calculated using the PBE and HSE06 functionals for (**a**) BC$_2$N, (**b**) BC$_2$P and (**c**) BC$_2$As monolayers. Charge densities of the valance band maximum (VBM) and conduction band minimum (CBM) orbitals are indicated on the top of the panel. Zero energy is set to the Fermi energy.

## Mechanical properties

And last but not least the mechanical stability of BC$_2$X (X = N, P, As) monolayers has been studied using the harmonic approximation method. Thus, we found out that all BC$_2$X considered are mechanically stable. The computations prove that BC$_2$X monolayers satisfy the elastic stability criteria^49^. The elastic constants are used to determine the other mechanical parameters such as: the bulk (B), shear (S), and Young’s (Y) moduli, Poisson’s ratio ($\nu$), and B/S ratio using the Voigt Reuss-Hill approximation^50^ (see Table 1). The bulk modulus refers to the ability of the monolayer to withstand compressing under applied force. The bulk modulus are determined to be: 14.63, 4.16, and 3.54 GPa for the BC$_2$N, BC$_2$P, and BC$_2$As, respectively. It is evident that the BC$_2$N monolayer can withstand greater compression as compared with the other monolayers. This result is supported by the calculation of the Young’s modulus, which is 22.81, 7.42, and 6.60 GPa for the BC$_2$N, BC$_2$P, and BC$_2$As, respectively. The shear modulus measures the resistance of the change to the structure, where the shear modulus increases as the material rigidity increases. The calculated S values are 9.20, 3.11, 2.77 GPa for X = N, P, and As respectively, which indicates that BC$_2$N monolayer has the highest rigidity within the members of this family. The B/S ratio measures the ductile or brittle nature of the material. We have determined the B/S is 1.59 (BC$_2$N), 1.35 (BC$_2$P) and 1.28 (BC$_2$As). Notice, that all BC$_2$X monolayers are brittle structures since the B/S value is less than 1.75^51^. Also, the calculation of the Poisson’s ratio, P, confirms the brittle behavior of the BC$_2$X sheets. We found out the values of P to be: 0.24, 0.20, and 0.19 for X = N, P, and As, respectively, i.e., all of them are less than 0.33^52^.

## Electronic properties

**Figure 3:**
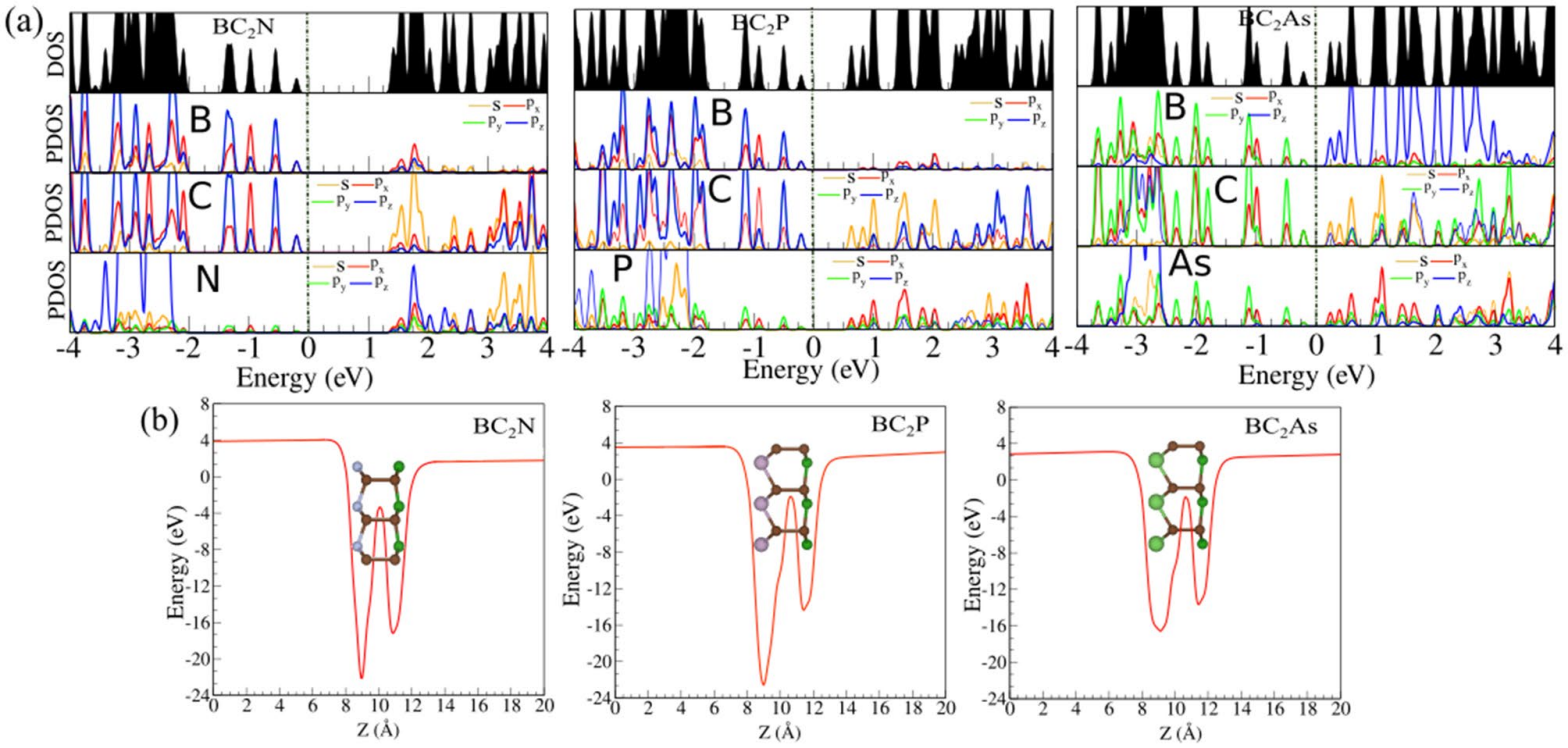
(**a**) Density of states (DOS) and partial DOS (PDOS) and (**b**) planar average electrostatic potential energy of BC$_2$N, BC$_2$P and BC$_2$As monolayers. The atomic structures are shown in the inset.

The calculated electronic band structures of the BC$_2$X (X = N, P, As) monolayers are shown in Fig. 2a–c. The charge densities of the valence band maximum (VBM) and conduction band minimum (CBM) orbitals are depicted in Fig. 2a–c (top parts). Our results demonstrate that BC$_2$N is an indirect semiconductor and has a bandgap of 1.51 eV using the PBE functional. However, the plane BC$_2$N has a direct experimental bandgap of $\sim$ 2 eV and a theoretical PBE-based bandgap of 1.6 eV, which is larger than the value of our BC$_2$N monolayer^37,38^. The VBM and CBM are located at the $\Gamma$ and M point, respectively. Similarly, BC$_2$N, BC$_2$P and BC$_2$As are semiconductors with indirect bandgaps of 0.78 eV and 0.39 eV, respectively according to the PBE method. Due to the underestimated bandgap values by the PBE-calculations, the HSE06^45^ hybrid functional was also utilized to evaluate the electronic band structure. The results obtained are depicted in Fig. 2a–c (blue curves). The bandgap value is calculated to be 2.68 eV using the HSE06 hybrid functional and the VBM and CBM are located at the $\Gamma$-point, resulting in a direct bandgap, which means the HSE06 approach changes the value and type of the bandgap of the BC$_2$N monolayer as compared with the PBE-based results. It is found out the HSE06 approach does not change the nature of the bandgaps of the BC$_2$P and BC$_2$As monolayers. The HSE06 values for the indirect bandgaps of BC$_2$P and BC$_2$As are 1.77 and 1.21 eV, respectively. The density of states (DOS) and partial DOS (PDOS) of the BC$_2$X monolayers are exhibited in Fig. 3a. From the DOS and PDOS of the BC$_2$N and BC$_2$P monolayers, the VBM is composed of the $p_{z}$ orbitals of B and C atoms, while the CBM comes from C-s orbital and $p_{x}$ orbital states of B (BC$_2$N) and P (BC$_2$P) atoms. In the case of the BC$_2$As monolayer, the VBM consists from $p_{y}$ of B and P orbitals states, while the CBM consists of B/C-$p_{z}$ and As-$p_{x}$ orbital. The average electrostatic potential energy of the BC$_2$X sheets is illustrated in Fig. 3b. From this, the work function was calculated using the following relation: $\Phi =E_{vacuum}-E_{F}$, where $E_{vacuum}$ is the vacuum energy (extracted from the electrostatic potential) and $E_{F}$ is the Fermi energy. The work function of the BC$_2$N, BC$_2$P and BC$_2$As monolayers is calculated as 4.32 eV, 4.00 eV and 3.50 eV, respectively, i.e., it decreases with the decrease of the X atom electronegativity (see Table 1).

## Optical properties

**Figure 4:**
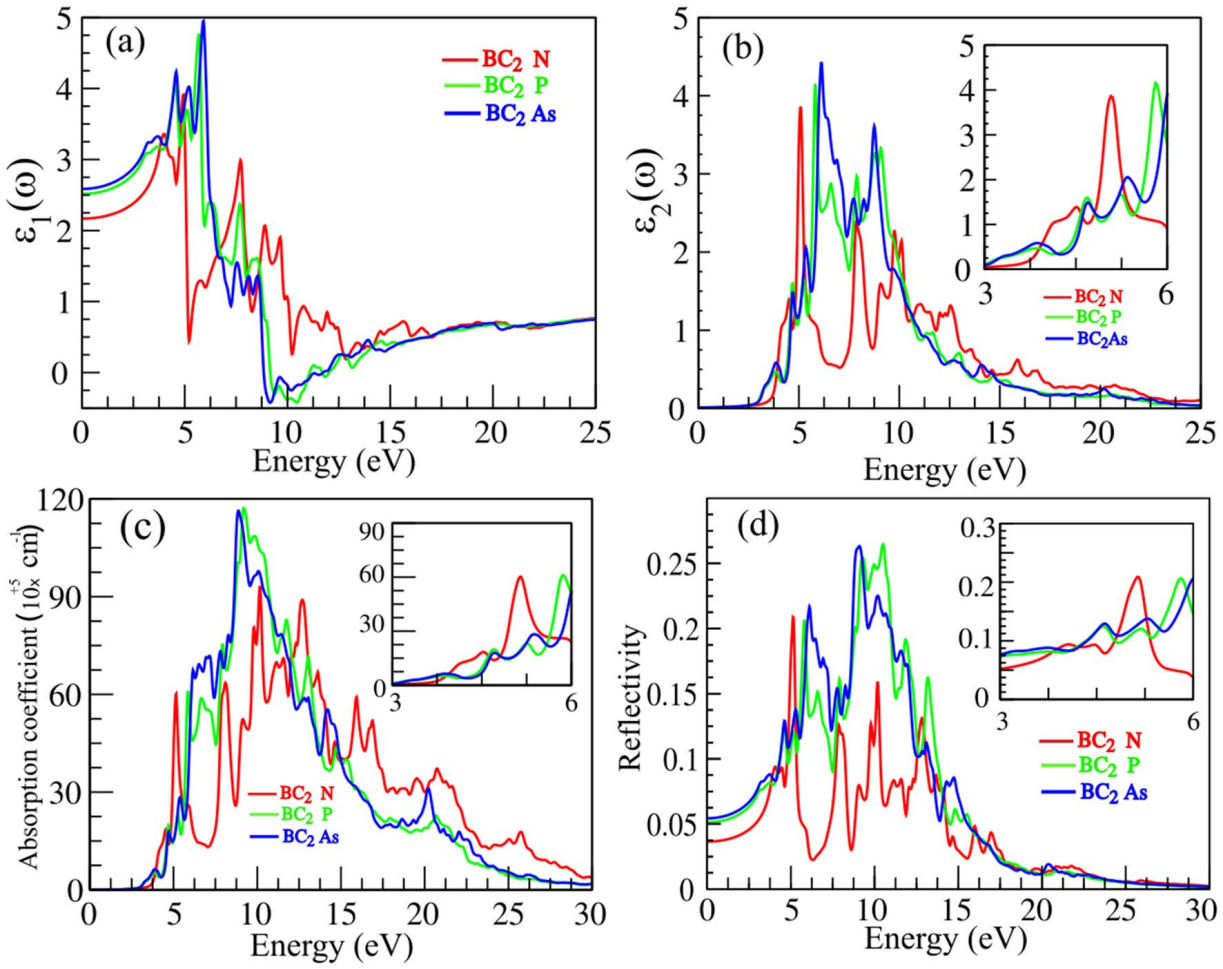
(**a**) Real and (**b**) imaginary parts of the complex dielectric constant, (**c**) absorption coefficient and (**d**) reflectivity of BC$_2$N, BC$_2$P and BC$_2$As monolayers.

To evaluate the possible optical applications of the discussed monolayer semiconductors, we calculated their most important optical parameters, namely, the complex dielectric functions of the monolayer materials which can be represented by: $\varepsilon (\omega )=\varepsilon _{1}(\omega )+i\varepsilon _{2}(\omega )$ where $\varepsilon _{1}$ and $\varepsilon _{2}$ are the real and imaginary terms, respectively. The imaginary part can be identified by using the random phase approximation (RPA), which counts the matrix elements of interband optical transitions between occupied and unoccupied states^53^. Once the imaginary part is obtained, by using the Kramers–Kronig relations^53^, the real part can be computed too. From the real and imaginary components, all other optical parameters of a material such as the absorption and reflectivity spectra can be calculated. Figure 4a–d illustrates the optical characteristics of the BC$_2$N, BC$_2$P and BC$_2$As monolayers. The real and imaginary parts are plotted in Fig. 4a,b. When looking at the complex dielectric function of a material, the static dielectric function $\varepsilon _{1}(0)$ and the high frequency dielectric function $\varepsilon _{1}(\infty )$ are important since these parameters describe the dielectric response to static and high energy electromagnetic fields, respectively. As seen in Fig. 4a, based on our computations, the values of 2.21, 2.50, 2.55 are obtained for the static dielectric functions of BC$_2$N, BC$_2$P and BC$_2$As monolayers, respectively. The high frequency dielectric function is calculated to be 3.85 (BC$_2$N), 4.12 (BC$_2$P) and 4.41 (BC$_2$As), meaning that these monolayers show a similar response to high energy electromagnetic field. Furthermore, as displayed in Fig. 4a,b, by increasing the energy of incident photons, both components of the dielectric function increase and exhibit the first peaks at 4.00 eV, 4.75 eV for BC$_2$N, 4.50 eV, 5.00 eV for BC$_2$P and 4.75 eV, 5.20 eV for BC$_2$As, respectively. The calculated absorption and reflectivity spectra are illustrated in Fig. 4c,d. Notably, the first main peaks of absorption appear at the energies of 5.00 eV, 5.75, 6.25 eV for the BC$_2$N, BC$_2$P and BC$_2$As monolayers respectively, while all monolayers show almost insignificant absorption properties in the low energy region of the electromagnetic spectrum. The first main absorption peak appears at approximately 5.00 eV for BC$_2$N, 6.00 eV for BC$_2$P and 7.50 eV for BC$_2$As. Finally, the first major reflectivity peaks occur at 5.00 eV for BC$_2$N, 6.00 eV for BC$_2$P and 7.00 eV for BC$_2$As with a reflectivity of about 21.5 %, 21 %, and 22 %, respectively. These results indicate that the 2D BC$_2$X materials proposed are transparent for visible light, however, are highly absorbing UV light.

### Band alignment

**Figure 5:**
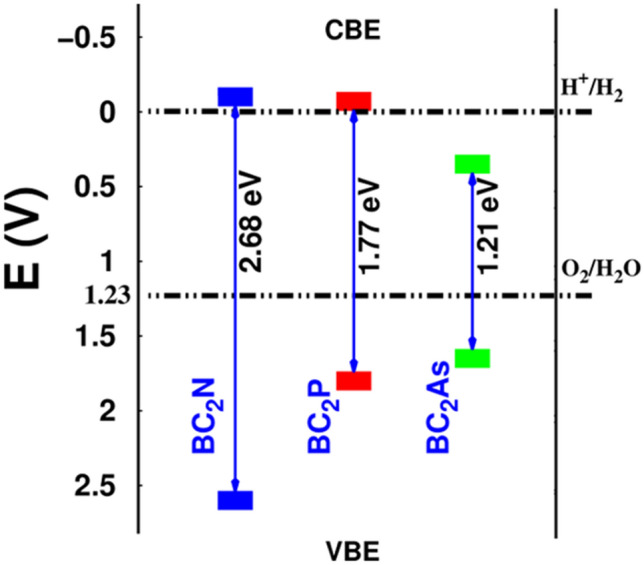
Band alignments of BC$_2$N, BC$_2$P and BC$_2$As monolayers, with respect to the normal hydrogen electrode potential (in Volts), for photocatalytic water splitting at pH = 0.

Figure 5 illustrates the band alignment of the oxidation potentials for water splitting with respect to the band edges of the BC$_2$N, BC$_2$P and BC$_2$As monolayers, obtained using the HSE06 with reference to the normal hydrogen electrode potential. Most importantly, BC$_2$N and BC$_2$P monolayers exhibit suitable bandgap values of $\sim$ 2.66 eV and 1.77 eV, respectively. The CB edge (CBE) and the VB edge (VBE) must be higher (more negative) and lower (more positive) than the hydrogen reduction potential of H$^{+}$/H$_{2}$ and the water oxidation potential of H$_{2}$O/O$_{2}$, respectively. The CBE is computed from the relation: $E_{CBE} = X - 0.5 E_{g} - 4.5$ eV^54–56^, and the VBE is $E_{VBE} = E_{CBE} + E_{g}$, where *X* is the geometric mean^57^ and 4.5 eV is the free electron energy with respect to the vacuum level. Obviously, the BC$_2$N and BC$_2$P monolayers would be advantageous photocatalysts for water splitting due to their suitable bandgaps and band edge alignments.

## Thermoelectric properties

Herein, the thermoelectric properties of the BC$_{2}$X (X = A, P, As) monolayers have been calculated using the semiclassical Boltzmann transport theory within the framework of the rigid band approximation. Electrical conductivity $\sigma$, Seebeck coefficient *S* and electronic thermal conductivity $\kappa$ are calculated using the following formula:

2 $$\begin{aligned} \sigma _{\alpha \beta }(T,\mu )= & {} \frac{1}{\Omega }\int \sigma _{\alpha \beta }(\epsilon )\left[ -\frac{\partial f_{0}(T,\epsilon , \mu )}{\partial \epsilon }\right] d\epsilon \end{aligned}$$

3 $$\begin{aligned} S_{\alpha \beta }(T,\mu )= & {} \frac{1}{eT\Omega \sigma _{\alpha \beta }(T,\mu )}\int \sigma _{\alpha \beta }(\epsilon )(\epsilon - \mu )\left[ -\frac{\partial f_{0}(T,\epsilon , \mu )}{\partial \epsilon }\right] d\epsilon \end{aligned}$$

4 $$\begin{aligned} \kappa ^{0}_{\alpha \beta }(T,\mu )= & {} \frac{1}{e^{2}T\Omega }\int \sigma _{\alpha \beta }(\epsilon )(\epsilon - \mu )^{2}\left[ -\frac{\partial f_{0}(T,\epsilon , \mu )}{\partial \epsilon }\right] d\epsilon \end{aligned}$$

where $\sigma _{\alpha \beta }(\epsilon )$ refer to the transport distribution tensor elements, which are calculated via Fourier interpolation of the band structure; $\Omega$, $\mu$ and *T* denote the cell volume, chemical potential and absolute temperature, respectively.

**Figure 6:**
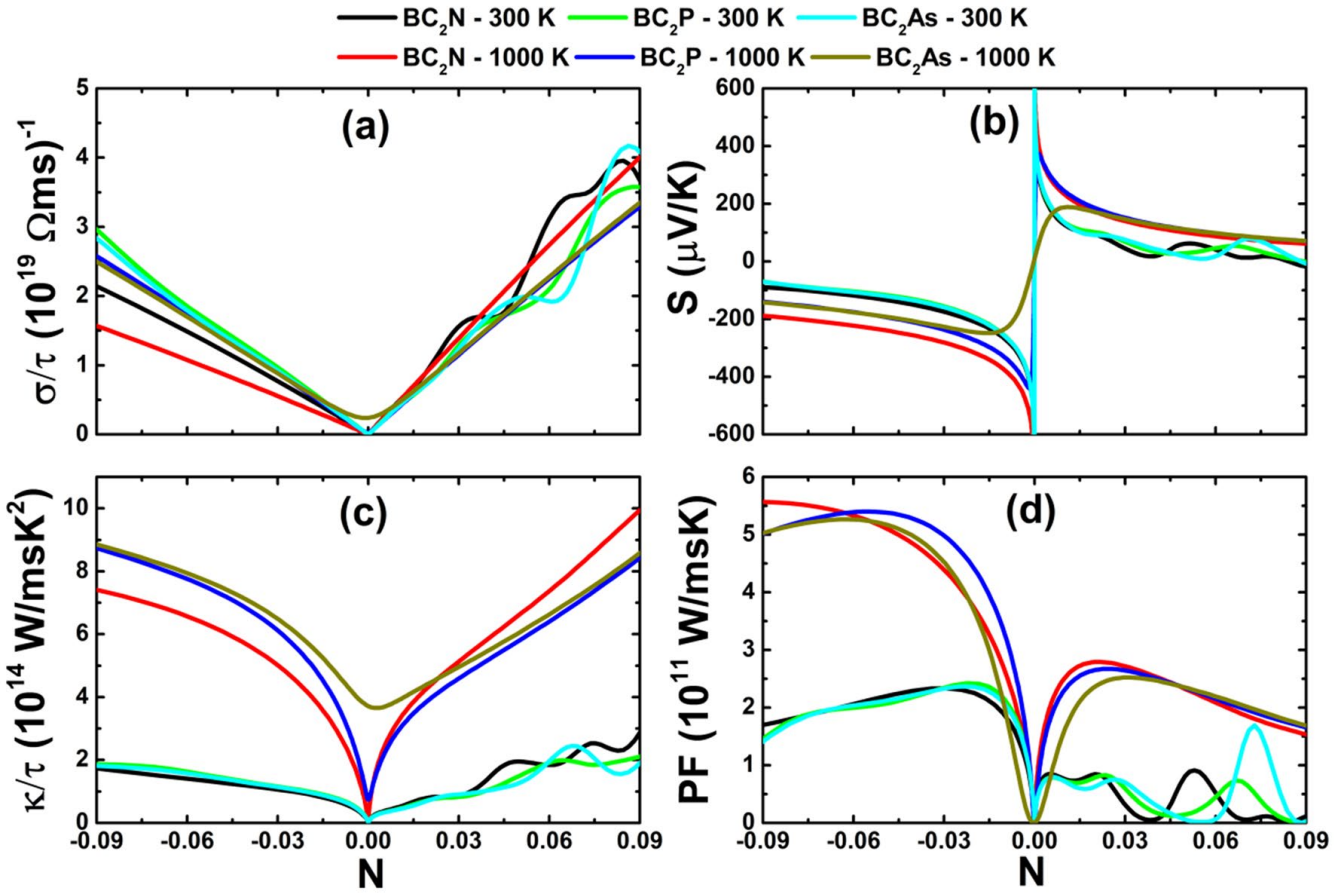
(**a**) Electrical conductivity, (**b**) Seebeck coefficient, (**c**) electronic thermal conductivity and (**d**) power factor of the BC$_{2}$X (X = N, P and As) monolayers at 300 and 1000 K.

Figure 6a depicts the electrical conductivity as a function of the doping level for the BC$_{2}$X monolayers at temperatures of 300 and 1000 K. Thermoelectric materials should have large electrical conductivity, which facilities the movement of charge carriers. Without doping, the electrical conductivity is quite small and it can be increased slightly by raising the temperature. The most notable increase is observed in the case of the BC$_{2}$As single-layer, for which a value of 0.24 $(10^{-19} \Omega \,{\text {ms}})^{-1}$ is reached. This transport parameter increases almost linearly with the doping level, however, at room temperature the slight fluctuation may be caused by the hole doping with a concentration larger than 0.038. For most of the considered doping levels, the BC$_{2}$P and BC$_{2}$As monolayers exhibit quite similar electrical conductivity values, which are larger (smaller) than that of the BC$_{2}$N monolayer in the *n*-region (*p*-region). Note, that the temperature increase may give rise to the reduction of this important parameter in the case of electron doping. When the systems are p-type doped, the temperature influences significantly only at high concentrations. It appears that the hole doping may be more favorable to create the charge carriers flow provided that the electrical conductivity is larger in the *p*-region than in the *n*-region at the same concentration of holes and electrons.

**Table 2:** Largest power factor (10$^{11}$ W/msK) of the BC$_{2}$X monolayers in the considered doping range from − 0.09 to 0.09.

	*n*-type		*p*-type		
	300 K	1000 K	300 K		1000 K
**BC**$_{2}$**N**	2.335	2.573	0.915		2.792
**BC**$_{2}$**P**	2.424	5.401	0.827		2.671
**BC**$_{2}$**As**	2.368	5.266	1.690		2.522

The Seebeck coefficient (thermopower) of the BC$_{2}$X monolayers is plotted in Fig. 6b. In thermoelectric materials, the temperature difference creates a potential that generates the electric force to push charge carriers. From the figure, one can see that large Seebeck coefficients are obtained for extremely small doping levels, with the largest values of 2555 (− 2636), 1329 (− 1429) and 632 (− 732) (μV/K) in *p*-region (*n*-region) for the BC$_{2}$N, BC$_{2}$P and BC$_{2}$As monolayer, respectively. By increasing the doping concentration, the thermopower decreases rapidly in the concentration range from − 0.03 to 0.03. Beyond this, the reduction rate becomes considerably smaller. Obviously, all three considered single-layers exhibit quite similar thermopower values at room temperature. Increasing the temperature to 1000 K may favor the electric potential evolution provided that the Seebeck coefficient increases notably, where the BC$_{2}$N shows the largest values. It is worth mentioning that at high temperature the thermopower of the BC$_{2}$As monolayer shows interesting behavior in the doping range from − 0.015 to 0.015, where it reaches maximum values of 188 (μV/K) (for N = 0.011) and − 249 (μV/K) (for N = − 0.015) in the *p*-region and *n*-region, respectively.

The doping level dependence of the electronic thermal conductivity for the BC$$_{2}$$X monolayers is illustrated in Fig. 6c for temperatures of 300 and 1000 K. In general, one can expect a similar variation of the electronic thermal conductivity $$\kappa _{el}$$ and electrical conductivity $$\sigma$$, provided by the proportional relation established by the Wiedemann–Franz law: $$\kappa _{el}~=~LT\sigma$$, where the Lorenz number and the absolute temperature are denoted by *L* and *T*, respectively. For good thermoelectric performance, low thermal conductivity is desired. Our simulations indicate that at room temperature the studied 2D materials exhibit quite similar electronic thermal conductivity values. This parameter is strongly dependent on temperature. For example, at extremely low doping level it increases from negligible values to 0.026 (BC$$_{2}$$N), 0.742 (BC$$_{2}$$P) and 3.715 (BC$$_{2}$$As) $$(10^{14}$$ W/msK$$^{2})$$ when increasing the temperature from 300 to 1000 K. At a high temperature and high doping levels, the BC$$_{2}$$N monolayer exhibits the smallest (largest) electronic thermal conductivity in the *n*-region (*p*-region), while those of the BC$$_{2}$$P and BC$$_{2}$$As monolayers show notable similarity.

Now we examine the electric power generation of the studied monolayers through the power factor parameter, which is deduced from the Seebeck coefficient and electrical conductivity as follows: $PF = S^{2}\sigma$. Larger power factors imply good ability for producing electricity from the temperature difference across a material. Figure 6d displays the power factor of the BC$_{2}$X monolayers. Note that for small charge carrier concentrations, this parameter is quite small, indicating poor electric production due to the low electrical conductivity. On increasing the doping level, this parameter shows a fast rapid increasing trend to reach its maximum before decreasing, with the exception of the hole doping at room temperature for which a significant fluctuation is observed. In Table 2 the largest power factor values for the considered doping levels are given. It appears that electron doping may favor more the electric productivity of the studied 2D materials than the hole doping.

## Conclusion

On the basis of first-principles investigation, we introduced BC$_2$N, BC$_2$P and BC$_2$As monolayers as novel 2D structures. The monolayers are proved to be stable as indicated by the calculations of the cohesive energy, phonon dispersion, AIMD simulation and elastic stability criteria. The mechanical study shows that all BC$_2$X monolayers have brittle nature. Also we found out the BC$_2$P monolayer can resist compression more than the other monolayers. The cohesive energy, the work function, and the bandgap values of BC$_2$X (X = N, P, and As) decrease as we move down the 5A group (in the periodic table), i.e., from N, P and As. The calculated bandgaps using the PBE (HSE06) for BC$_2$N, BC$_2$P and BC$_2$As monolayers are 1.51 (2.68) eV, 0.78 (1.77) eV and 0.39 (1.21) eV, respectively, which are appealing for applications in nanoelectronic devices. For optical applications, the BC$_2$X monolayers are 2D materials active in the ultraviolet spectral region. BC$_2$N and BC$_2$P can be good photocatalysts for water splitting. Our study further shows that the electrical conductivity of the BC$_2$X monolayers is very small at any temperature. Regarding the thermal conductivity, BC$_2$N has a small (large) electronic thermal conductivity in the *n*-region (*p*-region) with increasing temperature. Electron doping of the considered single-layers may be more favorable regarding electric power generation than hole doping, providing a larger power factor in the *n*-region than in the *p*-region. The novel BC$_2$N, BC$_2$P and BC$_2$As monolayers are promising materials for electronic, optical and energy conversion applications.

## Data Availability

The data that support the findings of this study are available from Bafekry but restrictions apply to the availability of these data, which were used under license for the current study, and so are not publicly available. Data are however available from the authors upon reasonable request and with permission of Bafekry.
